# Is the health-awareness of leaders related to the working conditions, engagement, and exhaustion in their teams? A multi-level mediation study

**DOI:** 10.1186/s12889-021-11985-1

**Published:** 2021-10-24

**Authors:** Luisa A. Grimm, Georg F. Bauer, Gregor J. Jenny

**Affiliations:** Center of Salutogenesis, University of Zurich, EBPI POH, Hirschengraben 84, 8001 Zurich, Switzerland

**Keywords:** Leadership research, Health-oriented leadership, Staff-care, Self-care, Exhaustion, Engagement, Job demands, Job resources

## Abstract

**Background:**

Research shows that leaders influence the health and well-being of employees, by being either a buffer or major source of employee’s work stressors. Various leadership behaviors and their relation to employee outcomes have been examined. Yet, a satisfactory explanation of how leaders’ behavior influences health has not been found. A new line of research investigates the construct of “health-oriented leadership”, that is, the health awareness of leaders towards themselves (“self-care”) and towards their employees (“staff-care”). It is hypothesized that this health-orientation has a direct effect on both leader’s and employees’ health, as well as an indirect effect mediated by their working conditions.

**Methods:**

Data were derived from four company research projects, that involved employee and leader surveys on work, health, and well-being. The sample consisted of 50 teams, with 191 leaders and 604 team members. To test the relation between a leader’s self-care and his/her engagement, exhaustion, as well as staff-care, multiple regression analyses and mediation analyses were conducted. The relation between a leader’s staff-care, the team’s job resources and demands, and the individual employee outcomes engagement and exhaustion were tested with multilevel analyses.

**Results:**

Regression analysis showed that the stronger a leader’s health-orientation towards him/−herself (“self-care”), the stronger was the health-orientation towards his/her employees (“staff-care”). A leader’s self-care was also associated with higher work engagement and lower exhaustion and this relation was mediated by his/her job resources and demands, respectively. Multilevel analysis showed that a leader’s staff-care was associated with employee work engagement and exhaustion, and that this relation was mediated by team-level job resources and demands, respectively.

**Conclusions:**

The health-orientation of leaders relates to their own as well as their teams’ engagement and exhaustion, which is partly mediated by job demands and resources. Thus the construct of health-orientation may prove worthy of further exploration. For practical conclusions, this study provides support for researching not different leadership styles with very specific facets, but a general orientation towards health, which can be implemented into coaching and consulting sessions for organizations.

## Background

The effect of leadership on employee health has been researched in many disciplines [1]. There is a consensus in the scientific community that leadership influences the health and well-being of employees [2], and that leaders have the potential to be either a buffer against work stressors [3] or be a major source of stress for employees [4, 5]. Research was conducted on various leadership styles and behaviors and their relation to employee outcomes, such as work engagement, stress, and health. Bass [4, 6] states that since 1980, there exists a leadership triad of transformational, transactional, and laisser-faire which cover the allegedly full range of leadership from the absence of leadership, to contingently rewarding followers accomplishments, and to inspiring followers towards higher goals that go beyond self-interest. Zimber and Gregersen [7] conducted a meta-analysis with 42 publications that deal with the relationship between leaders’ behavior and the state of health and well-being of their employees. They found that in particular, transformational and employee-orientated leadership are considered to be beneficial to health. The review by Nyberg et al. [8] and the meta-analysis by Judge and Piccolo [9] confirm a positive relationship between transformational leadership and job satisfaction. The meta-analysis by Montano, Reeske, Franke, and Hüffmeier [10] found that a high quality of leader-follower interaction is positively associated with mental health and destructive leadership is strongly negatively associated with mental health. Harms and Colleagues [11] found in a further meta-analysis that leader stress influences leader behavior and that leadership behaviors and leader-follower relationships are significant determinants of stress and burnout in employees. In the recent Version 11 of the International Statistical Classification of Diseases and Related Health Problems (ICD) of the World Health Organization, burnout is defined as a syndrome caused by stress at work that cannot successfully be processed, and is characterized by the three dimensions feeling of exhaustion, increasing negative attitude regarding the job, as well as a reduced ability of work performance [12]. In the ICD-11, burnout syndrome is therefore considered an occupational disease, which again underscores the importance of healthy work environments for organizations [13].

### The question about the core of healthy leadership

Yet, this previous research could not provide a satisfactory explanation of the question of how leaders’ behavior influences health [14]. Well-established, general leadership styles take health and well-being only indirectly into account [2, 10, 11, 15], and literature is thus criticized for being too unspecific when it comes to an explicit focus on health and well-being in leadership [16]. Both, various general leadership styles (such as transformational leadership) [2], and leadership constructs focusing on health and well-being [17], overlap theoretically and empirically. One possible explanation might be that not their differences, but common core aspects are important when it comes to health and well-being outcomes [2]. Thus, Nielsen and Taris [2] state that given the importance for employee (and organizational) functioning, it is crucial to direct future research towards the core of all those leadership styles, to find out what good leadership is, and how to promote it eventually. Meuser and Colleagues [18] address the same concern regarding leadership style research and counted up to 49 different leadership approaches and theories in published leadership research between 2000 and 2013. In their meta-analytic review, Harms and Colleagues [11] raise the same issue concerning leadership development and stress: “If perceptions of stress and reactions to ongoing stress are major drivers of leadership behaviors which, in turn, impact subordinate well-being and performance, it would seem that organizations concerned with leadership effectiveness now have evidence that leadership development could be done through addressing the stress factor”. Recent concepts of healthy leadership, for instance health-oriented leadership, explicitly consider health and well-being, not only in the constructs but also regarding outcomes, and are thus promising approaches for the explanations, prediction and influence of leaders on health and well-being [16, 17].

### Health-awareness of leaders

One possible new direction could be to investigate an explicit “health awareness” of leaders towards themselves and their employees. This line of research has investigated the role of leader health awareness on employee health, labeled “health-oriented leadership (HoL)” and “staff-care”, respectively [16]. This research involves the leaders as such in the process of creating healthy workplaces, as leaders not only have a major influence on the health of employees but are also exposed themselves to similar organizational burdens as their employees, especially lower and middle management [19]. In their research, Franke and Felfe also found that being aware of how work impacts one’s health (labeled “self-care”) is related to one’s own health [16]. Therefore, the HoL approach distinguishes between a leader’s self-care and his/her staff-care: Health awareness towards oneself is referred to as self-care - assessing how leaders deal with their own health [16]. Health awareness towards employees is referred to as staff-care and is assessed by leaders as a self-assessment [16]. Rephrasing Franke and Felfe’s [16] findings, if individuals are aware of their health and well-being, their resources and stressors, they are more likely to put more effort into its protection and promotion, which ultimately results in a better state of health and wellbeing [16]. A study by Franke, Felfe and Pundt [20] could show that HoL toward employees, as rated by employees, was positively related to well-being and negatively related to irritation, well-being complaints, and work-family conflict of employees.

HoL was developed in a largely deductive process [17] and describes the values of the leader in terms of an awareness of his or her own health and that of his or her employees, as well as behaviors such as effective health-related communication and the design of health-promoting working conditions [16]; it could therefore be understood as a common core concept underlying various leadership styles and behaviors. HoL is captured as a multidimensional construct and is composed of three subdimensions: health behavior, value of health, and health awareness [16, 21]. Health behavior includes personal lifestyle, and positive as well as negative health behaviors; value of health describes the attribution to health and health-promoting working conditions; health awareness includes perception, sensitivity, and reflection of health and its consequences. We focus on the awareness factor in this study, which can be addressed more directly through interventions compared to the very stable values and since awareness is a prerequisite for behavior change. Health awareness depicts a self-reflection that is necessary to achieve clarity and agreement on personal values, motives, and behaviors and, furthermore, to regulate actions toward oneself and others [21].

### Direct and indirect pathways of leadership on health

As shown above, most publications found a direct effect of leadership styles on employee health. But there is also copious evidence for indirect effects of leadership styles on employee health, which are mediated by working conditions or leader personality; for example, Jiménez and Colleagues [22] found an indirect effect of health-promoting leadership on stress and burnout mediated by employees’ resources. Gregersen and Colleagues [14] examined a range of publications and found that social support by leaders was particularly frequently examined as a potential resource. Moreover, the majority of publications confirmed a health-promoting effect of this type of support and largely supported the assumption that it can have both direct and indirect, e.g. buffering, effects on health. Also, leadership-related resources such as participation possibilities, recognition, appreciation, communication with leaders, and fairness were empirically confirmed. Furthermore, impatience, conflicts with leaders, pressure on employees, or other leadership deficiencies such as inadequate conflict management, were empirically tested and also confirmed as potential stressors. Magnavita, Tripepi, and Chiorri [23] could show in their study that intrusive leadership is associated with performance demands outside of normal working hours, high levels of stress, anxiety, and depression of employees; moreover, these effects were increased for employees that showed workaholism. Yet, it is important here to differentiate between work engagement and workaholism; workaholism describes an addiction to work which can be categorized as pathological, whereas work engagement describes healthy and positive thoughts and feelings towards work [23, 24]. Other studies examined leadership concepts that explicitly target well-being, such as health-promoting or health-oriented leadership. Research found that health-promoting leadership was negatively associated with burnout and perceived stress [25]. One study investigated the relationship between health-promoting leadership and employee recovery, perceived stress, and burnout and could show that health-promoting leadership is positively associated with recovery and negatively associated with perceived stress and burnout [22]. Regardless of whether well-being aspects are explicitly taken into account in the conceptualization of leadership, research currently shows that leadership is associated with health and well-being. This raises the question of what mechanisms help explain these direct relationships. Several studies examined the indirect effects of both general and health-promoting leadership on health and well-being by taking potential mediators into account. Skakon, Nielsen, Borg, and Guzman [15] found ample evidence in their systematic review of the indirect relationship between leadership and stress and employee affective well-being via improved working conditions. Jiménez et al. [22] found over and above the direct effect of leadership that employee resources mediate the relation of health-promoting leadership on stress and burnout. Another study found an indirect effect of “engaging leadership” on burnout and engagement, mediated by job demands and job resources; the author concludes that one reason why employee engagement increases through engaging leadership is because these leaders meet their employees’ basic psychological needs through inspiring, empowering and connecting them [26]. The importance of the relationship between job demands and job resources have also been discussed in the organizational setting [27]. These findings thus confirm that leadership behavior can act both as a direct or indirect resource or stressor.

### Health-aware leaders create resourceful and less stressful working environments

**Fig. 1:**
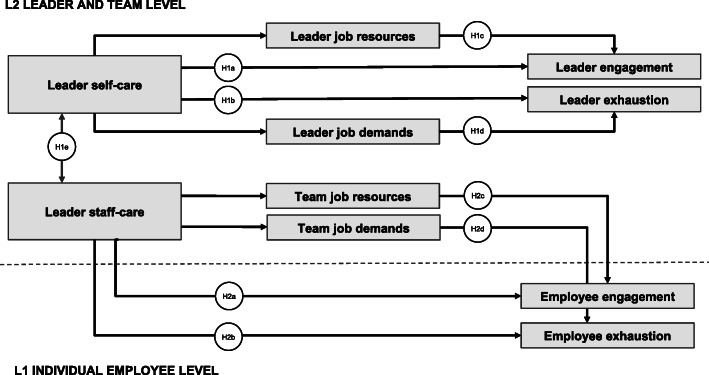
Overview of Hypotheses

Based on these considerations we aimed to investigate the direct and indirect role of leader health-awareness on employee health and well-being. A leader with strong “staff-care” will create a resourceful working environment for his/her employees and also try to lower potentially stressful job demands. Both of these will have an impact on employee outcomes such as exhaustion and work engagement [28]. Such a leader will also be aware of his/her own job demands and resources – in terms of “self-care” and consequently be less exhausted and more engaged at work. Thus, we derived the following hypotheses, which are illustrated in Fig. 1.

- *Study objective 1:* Is a leader’s self-care related to his/her engagement and exhaustion?
- *Hypothesis 1a*: A leader’s self-care is related to his/her engagement.
- *Hypothesis 1b*: A leader’s self-care is related to his/her exhaustion.
- *Hypothesis 1c*: The relation between a leader’s self-care and engagement is mediated by his/her job resources.
- *Hypothesis 1d*: The relation between a leader’s self-care and exhaustion is mediated by his/her job demands.
- *Hypothesis 1e*: A leader’s self-care is related to his/her staff-care.
- *Study objective 2:* Is a leader’s staff-care related to employee engagement and exhaustion?
- *Hypothesis 2a*: A leader’s staff-care is related to employee engagement.
- *Hypothesis 2b*: A leader’s staff-care is related to employee exhaustion.
- *Hypothesis 2c:* The relation between a leader’s staff-care and employee engagement is mediated by team-level job resources.
- *Hypothesis 2d:* The relation between a leader’s staff-care and employee exhaustion is mediated by team-level job demands.

## Methods

### Study design

This study was conducted with baseline data from a series of four research projects in companies between 2015 and 2019. Recruitment was done by the authors of this paper, as well as other researchers from their team (see also acknowledgements). Participants were recruited through mass mailings (such as newsletters), information events, social media postings and direct contact of company representatives. The projects comprised employee and leader surveys on work, health, and well-being that were followed by participatory optimization workshops. Projects 1 to 3 were conducted in medium and large-sized Swiss organizations in different sectors (cantonal police, mail-order pharmacy, public administration). Project 4 applied a self-directed digital approach to both surveys and workshops in a range of teams from different companies. In projects 1 to 3, leaders and staff received different questionnaires, with the questions having the same content but formulated according to their respective position. In project 4, leaders filled out questions in a digital coaching tool, while the team members filled out the questions in a separate online questionnaire. Hereby, no demographic data could be collected on employee level due to reasons of confidentiality. In all projects, participants voluntarily completed the questionnaires, guaranteeing their anonymity. No ethical review was necessary under national, university, or departmental rules (see Declarations section). The study was conducted under strict observation of ethical and professional guidelines.

### Study sample

Only teams were included where team leaders answered the questions. The response rates of the four studies were 67% (Police service), 72% (Mail-order pharmacy), 76% (Public administration) and 84% (Digital coaching). The resulting sample consisted of *N* = 50 teams, comprising *N* = 191 leaders, and *N* = 604 team members without supervisory function (see Table 1). The median was *M* = 11 employees per team. Among the leaders, 72.7% were male and 24.3% were female. The mean age was 45.94 (*SD* = 9.89). Among the team members (without sample 4 as no demographic data were collected of employees, see above), the sample consisted of slightly less male than females (44.1% vs. 55.9%), and the mean age was *M* = 39.32 years (*SD* = 11.66).

**Table 1:** Study sample

Companies	N Teams	N Leaders	N Team members	Total
Public administration teams	11	26	67	93
Police service teams	12	102	236	338
Mail-order pharmacy teams	6	42	121	163
Digital coaching teams	21	21	180	201
Total	50	191	604	795

#### Measurements

##### Leader self-care

Leader self-care was measured with an adapted version of the,Health-oriented Leadership scale’ [29]. The scale includes 6 facets (e.g. ‘I know which situations at work particularly stress me’ and ‘I notice early enough when things are getting too much for me’). The scale was measured with a five-point scale (“strongly disagree” to “strongly agree”). In the present study, Cronbach’s Alpha was *α* = .82.

##### Leader(s) staff-care

Leader’s staff-care was measured with an adapted version of the,Health-oriented Leadership scale (HoL)’ [29]. The scale includes 6 facets (e.g. ‘I know which situations at work particularly stress the employees’ and, I notice early enough when things are getting too much for the employees’). The scale was measured with a five-point scale (“strongly disagree” to “strongly agree”). In the present study, Cronbach’s Alpha was *α* = .81.

##### Job resources / job demands

Job resources were measured with the ‚Management Standards for work-related stress’- scales from the ‚Health and Safety Executive (HSE)’ [30], and supplemented by scales from the‚ Screening Activity Limitation and Safety Awareness Scale (SALSA) questionnaire‘ [31]. The factors are composed of 10 scales (7 job resources and 3 job demands) covered by 44 questions. Job resources are measured as a component of *control* (6 items, e.g. ‘I can decide when to take a break’), *role clarity* (4 items, e.g. ‘I am clear what is expected of me at work’), *managerial support* (5 items, e.g. ‘My line manager encourages me at work’), *peer support* (4 items, e.g. ‘I get help and support I need from colleagues’), *change transparency* (3 items, e.g. ‘Staff are always consulted about change at work’), *variety* (3 items, e.g. ‘There is something different to do every day’) and *possibilities for development* (3 items, e.g. ‘With this work, I can develop my abilities’). Latter two scales were derived from the SALSA questionnaire [31]. Job demands are measured as a component of *quantitative overload* (8 items, e.g. ‘I have to neglect some tasks because I have too much to do’), *negative relations* (4 items, e.g. ‘There is friction or anger between colleagues’), and *qualitative overload* (3 items, e.g. ‘There are things on this job that are too complicated.’). Latter scale is again derived from the SALSA questionnaire [31]. All items were rated on a five-point Likert-scale (‘strongly disagree’ to ‘strongly agree’). Reliability of all scales was good to very good (α = .73 to α = .89).

##### Engagement

Engagement, as the positive side of well-being, was measured with the ‚Utrecht Work Engagement Scale (UWES)’ [32] in the samples 1 to 3, and with one facet of engagement - positive activation measured by the ‚Positive Activation, Negative Activation and Valence Assessment Scale (PANAVA) scale ‘[33] in sample 4 (as in this sample, only this measure was available). The UWES scale includes 9 items (e.g. ‘At my work, I feel bursting with energy’). The scale was measured with a seven-point scale (“never” to “always”). In the present study, Cronbach’s Alpha of the UWES scale was *α* = .94. The PANAVA scale measures emotions in daily life via three factors (positive activation, negative activation, and valence). For measuring the included factor positive activation (which corresponds to engagement), the scale consists of the four adjective pairs ‘energy’, ‘wakefulness’, ‘motivation’ and ‘enthusiasm’ [33] representing the engagement dimension ‘vigor’ [34]. This subdimension was shown to be particularly associated with mental and physical health [35]. These four concepts were each rated on a seven-point semantic differential using pairs of words with opposite meanings (e.g., for energy from 1 = drowsy to 7 = energetic), asking the participant how they felt at work in the past few weeks. All items are rated on a seven-point continuum. Reliability of the PA scale was very good (α =. 83). In samples 1–3 for which both scales were available, correlation between the UWES scale and the PA scale was strong (r = .73, *p* < .001).

##### Exhaustion

Exhaustion, as the negative side of well-being and therefore counterpart of the engagement dimension, was measured with the,Copenhagen Psychosocial Questionnaire (COPSOQ) Scale ‘[36, 37]. The scale includes 4 items (‘How often have you felt worn out?’, ‘How often have you been physically exhausted?’, ‘How often have you been emotionally exhausted?’, and ‘How often have you felt tired?’). The scale was measured with a five-point scale (“never” to “always”), asking the participant how they felt at work in the past few weeks. In the present study, Cronbach’s Alpha was *α* = .89.

##### Control variables

By default, we included gender and age as control variables in all analyses on leader level.

#### Data analysis

Preliminary to hypothesis testing, we assessed the Intraclass Correlation Coefficients ICC(1) and ICC(2) for empirical justification of aggregating individual employee job resources and job demands on the team level. The ICC(1) value indicates the proportion of variance accounted for by group membership. A value of .01 might be considered as a small, .10 as a medium, and .25 as a large effect [38]. The ICC(2) value indicates the reliability of the group means, it has been suggested that cut-off values should be between .60 and .70 [39]. The ICC values suggested that aggregation on team level was justified (see Table 2) and thus group means were computed for both job demands and job resources. Further, where there was more than one leader of a team, group means of leader staff-care were computed for the respective team. Based on a median-split, each team was then assigned a dichotomous value for leader staff-care (low or high staff-care). This was done to enhance the reliability of the analysis, as leader staff-care is based on a single or a few leader self-assessments per team. All predictors on team level were then grand mean centered for cross-level analysis.

**Table 2:** Correlations between study variables

Variables	M	SD	ICC1	ICC2	2	3	4	5	6	7	8
*Leader level*											
1 Gender	1.27	0.45	–	–	−.28**	.06	−.09	−.02	.16*	.05	.08
2 Age	49.77	10.14	–	–	–	−.06	.10	.06	−.26**	−.01	−.11
3 Self-care	4.13	0.51	–	–	–	–	.46**	.31**	−.32**	.41**	−.25**
4 Staff-care	3.83	0.48	–	–	–	–	–	.18*	−.25**	.34**	−.22**
5 Engagement	5.23	0.97	–	–	–	–	–	–	−.44**	.52**	−.29**
6 Exhaustion	2.56	0.79	–	–	–	–	–	–	–	−.25**	.43**
7 Job Resources	3.85	0.47	–	–	–	–	–	–	–	–	−.35**
8 Job Demands	2.20	0.46	–	–	–	–	–	–	–	–	–
*Employee level*											
5 Engagement	4.97	1.15	0.09	0.57	–	–	–	–	−.52**	.57**	−.40**
6 Exhaustion	2.62	0.83	0.14	0.66	–	–	–	–	–	−.38**	.54**
7 Job Resources	3.78	0.57	0.31	0.82	–	–	–	–	–	–	−.47**
8 Job Demands	2.08	0.50	0.18	0.72	–	–	–	–	–	–	–

*Note*. * *p ≤* .05, ** *p ≤* .01 (two-tailed), *N* leaders = 181–189; *N* employees = 601–603, *M* = Mean, *SD* = Standard Deviation, *ICC =* Intraclass Correlation

##### Leader level analysis

To test the relation between leader self-care and the leader outcomes engagement, exhaustion and staff-care (hypotheses 1a, 1b, 1e), we conducted three multiple regression analyses with all variables at level 2. In each regression model, age and gender were added as control variables. To test if job resources and job demands mediated the above-postulated relations between leader self-care and engagement and exhaustion, respectively (hypotheses 1c, 1d), direct and indirect effects were tested for significance using bootstrapping and confidence intervals (process procedure for SPSS Release 2.16.3, Hayes, 2013).

##### Cross-level analysis

To test the relation between leader staff-care, team job resources and demands, and the individual employee outcomes engagement and exhaustion (hypotheses 2a-2d), we employed two series of multilevel analyses with leader’s staff-care and team job resources and demands, respectively, at level 2 and the individual employee outcomes (engagement and exhaustion) at level 1. For each analysis, we compared three models starting with the null model that includes only the intercept. Then, in model 1, leader staff-care (low/high) was included, and in model 2, team job resources or demands were added. The improvement of the model can be compared by using the Akaike information criterion (AIC) on a smaller-is-better-basis. The significance level for all analyses was set at *p ≤* .10 to guard against type II error due to the small sample size at level 2. The Monte Carlo method recommended by Selig and Preacher (2008) was used to estimate confidence intervals for the hypothesized cross-level 2–2-1 mediation effects (hypotheses 2c and 2d). All analyses were conducted with SPSS 25 for Mac.

## Results

Table 2 illustrates the results of the intercorrelations on leader and employee level and the aggregation analysis of the variables. The aggregation analysis showed that all *ICC(1)* values were statistically significant and ranged between .09 and .31 and revealed good intraclass correlation scores. The same pattern can be seen in the ICC(2) (range: .57–.82). Thus, it is to conclude that there is sufficient empirical justification for aggregating the individual-level variables on the team-level. All correlations between the mediators and outcomes revealed on both levels significant relations and directions that were expected (i.e., positive correlations between job resources and engagement and negative correlations between job demands and exhaustion).

### Results from leader level analysis

We conducted linear regression analyses to test Hypotheses 1a, 1b and 1e. The results of these associations between leader self-care and respective leader outcome are shown in Table 3. Hypothesis 1a states that a leader’s self-care is related to his/her engagement. The first regression model examined the relation between leader self-care and leader engagement as outcome variable. The results showed that leader self-care positively predicted leader engagement (*β* = 0.66, *p* < .001), confirming hypothesis H1a. Hypothesis 1b states that a leader’s self-care is related to his/her exhaustion. The second regression model examined the relation between leader self-care and leader exhaustion as outcome variable, and the results showed that leader self-care negatively predicted the leader exhaustion (*β* = − 0.48, *p* < .001), confirming hypothesis H1b. Finally, Hypothesis 1e states that a leader’s self-care is related to his/her staff-care. The third regression model examined the relation between leader self-care and leader staff-care as outcome variable. The results showed that leader self-care positively predicted the leader’s staff-care (*β* = 0.44, *p* < .001), confirming hypothesis H1e.

**Table 3:** Associations between leader self-care and leader outcome (three separate regression models; the control variable age and gender are not depicted)

*Variables*	*B*	*SE B*	*Beta*	*R*^*2*^	95% CI
M1 Leader self-care → Leader engagement	0.657*	0.141	0.338*	0.114*	[.38, .94]
M2 Leader self-care → Leader exhaustion	−0.484*	0.111	0.413*	0.171*	[−.71, −.27]
M3 Leader self-care → Leader staff-care	0.444*	0.061	0.503*	0.253*	[.32, .57]

*Note*. † *p ≤* .10, * *p ≤* .05 (two-tailed), *N* (M1) = 173, *N* (M2) = 173, *N* (M1) = 171, *B =* Un-standardized Coefficients, *SE B =* Standard Deviation of *B*, *Beta =* Standardized Coefficients, *CI =* Confidence Intervals

**Fig. 2:**
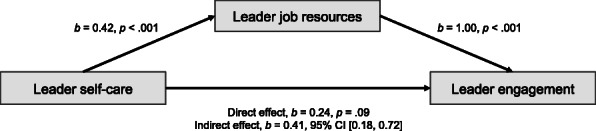
Mediation analysis of leader self-care, job resources, and engagement

**Fig. 3:**
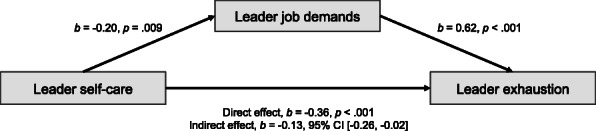
Mediation analysis of leader self-care, job demands, and exhaustion

We conducted mediation analyses with the PROCESS procedure [40] to test Hypotheses 1c and 1d. Hypothesis 1c states that the relation between a leader’s self-care and engagement is mediated by his/her job resources, and Hypothesis 1d states that the relation between a leader’s self-care and exhaustion is mediated by his/her job demands. Figure 2 reveals the significant indirect effect of leader’s self-care on leader engagement through leader job resources, *b* = 0.41, BCa 95% CI [0.18, 0.72]. Figure 3 reveals the significant indirect effect of leader’s self-care on leader exhaustion through leader job resources, *b* = − 0.13, BCa 95% CI [− 0.26, − 0.02]. These results from mediation analyses showed that there was a significant indirect effect for leader self-care on leader engagement through leader job resources, and for leader self-care on leader exhaustion through leader job demands, confirming both hypotheses H1c and H1d.

### Results from cross-level analysis

Tables 4 and 5 summarize the results of Hypotheses 2a-2d. We applied multilevel analysis to examine the direct impact of leader staff-care on individual employee engagement, as well as the indirect impact mediated by team job resources. Similarly, we examined the direct impact of leader staff-care on individual employee exhaustion, as well as the indirect impact mediated by team job demands.

**Table 4:** Multilevel analysis of level 2 variables (leader staff-care, team job resources) on level 1 employee engagement

	Null Model	Model 1	Model 2
	***B*** (SE)	***B*** (SE)	***B*** (SE)
Intercept	4.94 (0.07)***	4.94 (0.07)***	4.93 (0.06)***
Leader staff-care (low/high)		0.22 (0.13)†	−0.04 (0.12)
		[−0.04, 0.50]	[−0.30, 0.20]
Team job resources			0.98 (0.19)***
			[0.61, 1.37]
Variance within groups	1.19 (0.07)***	1.19 (0.07)***	1.18 (0.07)***
Variance between groups	0.11 (0.04)**	0.10 (0.04)*	0.04 (0.03)
AIC	1859.54	1858.86	1839.35

Note. † p ≤ .10, * p ≤ .05, ** p ≤ .01 ***p ≤ .001 (two-tailed), [95% Confidence Intervals],

*N* = 604, *B =* Estimate of Fixed Effects, *SE =* Standard Error, *AIC =* Akaike Information Criterion

**Table 5:** Multilevel analysis of level 2 variables (leader staff-care, team job demands) on level 1 employee exhaustion

	Null Model	Model 1	Model 2
	***B*** (SE)	***B*** (SE)	***B*** (SE)
Intercept	2.63 (0.06)***	2.63 (0.05)***	2.64 (0.04)***
Leader staff-care (low/high)		−0.22 (0.11)*	−0.12 (0.09)
		[−0.44, − 0.01]	[− 0.29, 0.05]
Team job demands			1.02 (0.18)***
			[0.65, 1.38]
Variance within groups	0.59 (0.04)***	0.59 (0.04)***	0.59 (0.04)***
Variance between groups	0.09 (0.03)***	0.08 (0.03)**	0.03 (0.02)*
AIC	1445.69	1443.60	1424.03

Note. † p ≤ .10, * p ≤ .05, ** p ≤ .01 ***p ≤ .001 (two-tailed), [95% Confidence Intervals],

*N* = 604, *B =* Estimate of Fixed Effects, *SE =* Standard Error, *AIC =* Akaike Information Criterion

Table 4 summarizes the results of Hypothesis 2a and 2c. Hypotheses 2a states that a leader’s staff-care is related to employee engagement. The level 2 predictor staff-care was included first. Model 1 shows that leader staff-care was marginally positively related to individual employee engagement (*B* = 0.22, *p* = .100). These results showed that Hypothesis 2a can be cautiously confirmed. We then tested Hypotheses 2c whether the relation between a leader’s staff-care and employee engagement is mediated by team-level job resources. Model 2 in Table 4 shows that team job resources are significantly related to individual employee engagement (*B* = .98, *p* = .000), and that leader staff-care is no longer significant (*B* = −.04, *p* = .724), confirming Hypothesis 2c. Also, Model 2 had the smallest AIC value of all the models and was to be considered as the best-fitting model. This indirect effect from leader staff-care via job resources on employee engagement was estimated by the Monte Carlo method [41] to lie between 0.14 and 0.44 on with 99% confidence. Since none of these confidence intervals include 0, it can be concluded that the relation between leader staff-care and employee engagement was mediated by employee job resources.

Table 5 shows the results of Hypothesis 2b and 2d. Hypotheses 2b states that a leader’s staff-care is related to employee exhaustion. The level 2 predictor staff-care was included first. Model 1 shows that leader staff-care was negatively related with individual employee exhaustion (*B* = − 0.22, *p* = .043). Based on the results, Hypotheses 2b can be confirmed. We then tested Hypotheses 2d whether the relation between a leader’s staff-care and employee exhaustion is mediated by team-level job demands. Model 2 in Table 5 shows that team job demands are significantly related to individual employee exhaustion (*B* = 1.02, *p* = .000), and that leader staff-care is no longer significant (*B* = − 0.12, *p* = .172), confirming Hypothesis 2d. Again, Model 2 had the smallest AIC value of all the models and was to be considered as the best-fitting model. The indirect effect from leader staff-care via job demands on employee exhaustion was estimated by the Monte Carlo method [41] to lie between − 0.20 and − 0.06 with 99% confidence. Since none of these confidence intervals include 0, it can be concluded that the relation between leader staff-care and employee exhaustion was mediated by employee job demands.

## Discussion

The main intention of this study was to investigate the relationships between the health-orientation of a leader towards him- /herself (being aware what disturbs and motivates themselves) and leader well-being (operationalized by leader engagement and exhaustion).; as well as the health-orientation of a leader towards his or her employees (being aware what disturbs them and what motivates them) and employee well-being (operationalized by employee engagement and exhaustion). In addition to examining direct associations, we also examined indirect pathways, including job demands and job resources as mediators. Specifically, the study addressed two study objectives with corresponding hypotheses:

First, we wanted to examine whether a leader’s self-care is related to his/her engagement and exhaustion, and if job demands and resources have an additional effect on these relations. The results illustrate that the stronger a leader’s health-orientation towards him/−herself (‘self-care’), the stronger is the health-orientation towards his/her employees (‘staff-care’). On leader-level, the results showed that a leader’s self-care is associated with higher work engagement and lower exhaustion and that this relation is mediated by his/her job resources and demands, respectively.

Second, we addressed the question if a leader’s staff-care is related to employee engagement and exhaustion, and again, whether job demands and resources have an additional effect on these relations. The cross-level results showed that a leader’s staff-care is associated with employee work engagement and exhaustion and that this relation is mediated by team-level job resources and demands, respectively.

Based on the results of the study, the health-orientation of a leader is a promising construct worthy to be further explored. With regard to previous research that highlighted the missing of a consistent definition and operationalization of the concept of leadership [14] and the issue of multiple leadership behaviors and styles that intercorrelate [2], this study contributes to a new direction on leadership research and health. It intended to focus on a potential core concept of leadership, that is, leader health-orientation, which might be a crucial explanatory factor regarding employee health and well-being. Our results are consistent with the findings of Jiménez et al. [22], according to whom health-promoting leadership is positively related to recovery and negatively related to perceived stress and burnout. Our results also reflect the proposition by Rudolph and colleagues [17], in which components like attitudes of healthy leadership influence well-being outcomes, operationalized in our study by leader health-orientation. Our study could confirm the findings mentioned above by Franke, Felfe and Pundt [20]. In line with directives of previous research, we included mediating variables that play a role in explaining the relationship between leadership and employee health and well-being; our study confirmed findings from several studies regarding additional indirect effect of the relationship of leadership on (employee) well-being through several variables, for example, working conditions [22, 26]. Thus, this study contributes a new perspective on the multifactorial relationships between leadership and health at work [14].

### Strengths and limitations

The crucial strength of the paper is the application of a multilevel approach to the study design and statistical analysis, connecting the leaders’ assessments of their staff-care with the employees’ assessments of their health and well-being. Thus, the analysis allowed to take effects of variables measured independently at different levels into account and respected the nature of organizational structures, that an employee’s individual health and well-being may be dependent on his/her team affiliation and leader, respectively. Therefore, worthy of attention is how to improve a leader’s health-awareness and to promote job resources at the team level to improve work engagement at the micro-level, thereby enhancing a healthy workplace. To interpret organizational study results and derive recommendations for action in practice, such multilevel analysis is one crucial aspect.

However, there are some limitations in this research, which should be taken into consideration for future studies. As data collection was conducted on a cross-sectional basis, the study is prone to predictive limitations. Because predictor and outcome are assessed at the same time, there is no direct evidence of a causal relationship that is needed to draw predictive conclusions based on the differences found [42]. The study is also based on the leader’s self-assessment of his/her staff-care. Often self-assessment and external assessment differ from each other. Combining the two perspectives of a leader’s health orientation – i.e. the leader perspective and the team perspective – could bring us closer to a more comprehensive picture of ways in which leaders can promote employees health [20, 43]. Similarly, the analyses that take the employee-level into account, are also based on self-reports. Self-report studies do have advantages (e.g. inexpensive, long reach, shorter time frame), but they also tend to have disadvantages compared to other, objective measures. People are oftentimes biased when reporting on their own experiences, an example of which is social desirability. A problem could also be that people have a different interpretations of the questions and the range of rating scales. Therefore, future research should focus on combining subjective self-report measures with objective measures, such as turn-over rates or third-party evaluation. Additionally, the sample size of 50 teams could be a limitation. This is not the case for the leader level analyses, but might be for the cross-level analyses. However, finding relationships in a small sample illustrate large effect sizes [44]. Another critical point is that in our study, psychological well-being (engagement and exhaustion), was assessed, yet no data on the physical health status of the leaders was collected. Therefore, no statements can be made regarding any implications the physical health status might have, as leaders might differ in their perceptions on their own, as well as their employee’s status of health, depending on whether leaders themselves are in good or ill health. Finally, this study is based on a heterogenous sample, consisting of the full range of employees in a cantonal police corps, in a mail-order pharmacy, in a public administration (study samples 1–3), as well as various company sectors and sizes in study sample 4 – providing for good generalizability of the results. Still, further studies based on more homogenous samples would allow for more in-depth analysis of leader-follower dynamics in different sectors (for example, in the health care sector) and the typical contextual factors (such as organizational structure, hierarchy, readiness for change [14, 15, 45–47] that are potentially relevant.

## Conclusions

This study leads to several theoretical and practical conclusions. First, previous research of leadership and health focused on the differences of leadership styles, although a great number of these leadership styles and behaviors intercorrelate [1]. Therefore, the added value of this previous research, especially regarding its transfer into practice, is not clear. It seems to be of importance to rather focus on what lies at the core, which might be a certain health-orientation of leaders, to foster healthy, prosperous, and performative work environments. For practical conclusions, this study provides support for researching a general orientation of leaders towards health, which can then be implemented into coaching and consulting sessions for organizations.

## Data Availability

The data was collected through the research group named in the affiliations by means of anonymous and voluntary employee surveys in the companies participating in the study. The data was not publically available before this study. The datasets used and/or analyzed during the current study are available from the corresponding author on reasonable request.

## Abbreviations

*ICD, ICD-11*: International Classification of Diseases 11th Revision
*HoL*: Health-oriented Leadership
*HSE*: Health and Safety Executive
*SALSA*: Screening Activity Limitation and Safety Awareness Scale
*PANAVA*: Positive Activation, Negative Activation and Valence Assessment Scale
*UWES*: Utrecht Work Engagement Scale
*COPSOQ*: Copenhagen Psychosocial Questionnaire
*ICC*: Intraclass Correlation Coefficient
